# Biophilic design, neuroarchitecture and therapeutic home environments: harnessing medicinal properties of intentionally-designed spaces to enhance digital health outcomes

**DOI:** 10.3389/fmed.2025.1610259

**Published:** 2025-07-10

**Authors:** Grzegorz Bulaj, Maria Forero, Dorothy Day Huntsman

**Affiliations:** ^1^Department of Medicinal Chemistry, College of Pharmacy, University of Utah, Salt Lake City, UT, United States; ^2^OMNI Self-Care, LLC, Salt Lake City, UT, United States; ^3^Seed Healthcare, Miami, FL, United States; ^4^Click Therapeutics, New York, NY, United States; ^5^Accelmed, Miami, FL, United States; ^6^MF7 Ventures, Miami, FL, United States; ^7^Dayhouse Studio, Salt Lake City, UT, United States

**Keywords:** prescription digital therapeutics, mobile medical app, virtual reality, built environment, household, housing, salutogenic, biophilia

## Abstract

Digital health technologies (DHT) support patient-centered care by delivering behavioral, educational, self-efficacy and self-management interventions. Yet, multifactorial chronic diseases are shaped by complex interactions between genetics, environment and behavior, embodied in social and commercial determinants of health. Given that people in the United States spend on average 18 h per day at home, the impact of home environment on a person's health is underutilized in medicine. Herein, we discuss opportunities to improve therapy outcomes through bridging digital interventions with intentionally-designed restorative and multisensory environments that simultaneously foster physiological and emotional homeostasis. Harnessing positive effects of biophilic design, neuroarchitecture and therapeutic home environments can enhance the effectiveness of digital interventions, including digital therapeutics (DTx), wearables and drug + digital combination therapies that utilize “prescription drug use-related software” (PDURS) framework. Real-world barriers to advance these solutions include a lack of public awareness about connections between the built environment, health and wellbeing, the knowledge gap in long-term clinical outcomes of biophilic interventions, and a limited funding for advancing “biophilic design as an adjunctive therapy” applications. In conclusion, creating digital health ecosystems that favor symbiosis between digital health interventions and enriched environments can promote sustained behavior change, elevate precision care and improve value-based healthcare outcomes.

## 1 Introduction

DHT belong to a broad category of mobile devices and software that deliver clinical benefits through mobile and virtual reality (VR) apps, video games, digital health platforms, wearables and medical devices. A rapid growth of DHT includes an emergence of digital therapeutics (DTx), or Software as a Medical Device products, intended to diagnose and treat medical conditions (1, 2). An example of DHT supporting patient care are digital health platforms that provide medication management, monitoring symptoms and other disease management services (3). Digital interventions and pharmacotherapies can be integrated using “prescription drug use-related software” (PDURS) framework or as adjunctive DTx in combination with drugs intended to treat specific conditions (4). Adoption of DHT in medicine is limited by reimbursement rates, regulatory policies, and concerns from diverse healthcare stakeholders (5–8). Challenges for DHT include patient engagement and attrition that can impact long-term therapy outcomes (9–11). Many mobile apps for chronic diseases that are available in Google Play Store and Apple App Store lack the acceptable standards for the quality and content, undermining their effectiveness (12).

A majority of chronic conditions are multifactorial diseases where both the gene-environment interactions and a patient's behavior play important roles in their etiology and treatment outcomes (13). Environmental exposure has a significantly greater influence on non-communicable chronic diseases than genetic predisposition (14). For example, the presence of natural environments, e.g., neighborhood green spaces, can positively impact mental and physical health through physical and social activities (15–17). The intersection of housing and health is recognized as a means for public health interventions (18, 19), as well as to improve patient outcomes (20–22).

The concept of intentionally-designed environments that promote health is embodied in biophilic design, salutogenic architecture and neuroarchitecture. Biophilic design is an approach to improve health and wellbeing by incorporating natural elements into the built environment (23). Salutogenic architecture aims to create health-promoting spaces by supporting a person's “sense of coherence” (24), while neuroarchitecture is focused on how architectural features influence the human brain, cognitive functions, emotions and behavior (25). Grounded in biophilic design and the self-care model, we recently described the therapeutic home environments intended to provide clinical benefits for people with chronic conditions (20, 26). In this work, we discuss a confluence of design and digital health, as we highlight an opportunity to bridge behavior change interventions with living spaces that support homeostasis and lifestyle medicine. The thesis of this perspective article is that the effectiveness of DHT can be improved when a patient lives in an intentionally-designed home environment that fosters health and wellbeing.

## 2 Digital health offers multimodal therapies plus at-home convenience

DHT aim to improve health outcomes by delivering diverse therapeutic modalities, including behavioral and cognitive therapies (e.g., cognitive behavioral therapy, CBT, or acceptance and commitment therapy), mindfulness, health education, physical therapy, disease self-management, remote patient monitoring, coaching, relaxation techniques and biofeedback, among many others (27). These interventions are provided with at home convenience, as exemplified by digital health platforms for chronic disease management, such as Welldoc and Dawn Health, or a prescription digital therapeutic, RelieVRx, an FDA-authorized VR technology for patients with a moderate to severe chronic low back pain (28, 29). Clinical benefits of digital interventions span a wide range of chronic conditions, e.g., cancer (30), neurological (31), neurodegenerative (32), mental (33), metabolic (34), cardiovascular, and autoimmune disorders (35). The breadth, depth, flexibility, accessibility and scalability of multimodal DHT to treat chronic diseases make digital interventions an attractive value proposition for healthcare stakeholders.

DHT are also gaining popularity through fitness trackers and other consumer health wearables that enable monitoring vital signs and disease symptoms (36, 37). Wearables such as Apple Watch, Oura Ring, FitBit, Garmin Health, Samsung Galaxy watch are merging with medical device functionalities, based on receiving the FDA authorization or clearance for specific applications. These aforementioned wearables and their associated apps promote lifestyle medicine (e.g., physical activities, sleep hygiene, stress management), while ongoing research points toward their applications in the prevention of chronic conditions, such as irritable bowel syndrome or mild cognitive impairment (38, 39). An apparent advantage of wearables coupled with mobile apps is their ability to bridge daily activities with biofeedback and health outcomes (40).

Innovation of medical treatments includes integration of DHT with pharmacotherapies, leading toward drug + digital combination therapies (41–43). The indication-specific Rx+DTx combinations are illustrated by adjunctive DTx such as reSET-O app (in combination with buprenorphine for opioid use disorder), and Rejoyn app (in combination with antidepressant drugs for the treatment of major depressive disorder). The FDA's guidelines on prescription drug use-related software represent a paradigm shift in evolving medical treatments by enabling integration of Rx and DTx through drug labeling (44). Currently, companies like Click Therapeutics (USA), Remepy (Israel) and Closed Loop Medicine (UK), advance the development of personalized drug + digital combination therapies (aka “software-enhanced drugs,” or “hybrid drugs”) for migraine, Parkinson's disease, cancer and other indications. As discussed below, DHT are positioned to integrate pharmacological and behavioral interventions with a patient's home environment that promotes healing and the tertiary prevention.

## 3 Health behavior is a function of a person and environment

Grounded in diverse theories, health behavior includes beliefs, motivation, abilities and daily actions that support health and prevent diseases (45). Most digital interventions targeting health behavior change utilize goal setting and self-management (46). However, the impact of housing environment on health behavior has been largely overlooked, perhaps with an exception for trauma-informed care. For people experiencing post-traumatic stress disorder, trauma-informed design creates spaces intended to promote relaxation and feeling safe, while removing adverse environmental stressors (47). On the other hand, extensive research on migraine triggers, such as stress, disrupted sleep, lighting and air pollution, has not translated to studying migraine-informed home spaces that support lifestyle modifications and prevent headaches (20, 26, 48–50). Among general population, housing conditions that lead to sleep disruption can negatively impact mental health (51).

The built environment that promotes health behavior is likely to prevent chronic diseases (52, 53). Based on the Kurt Lewin's theory that behavior is a function of a person and environment, Brug and colleagues studied associations between homes, schools and workplaces (defined as micro-environment) and behaviors leading to obesity and cardiometabolic conditions (54). In another study, the relationships between the exposome and risks for developing diabetes revealed that neighborhood walkability and greenspaces can reduce the risk for type 2 diabetes (55).

**Figure 1:**
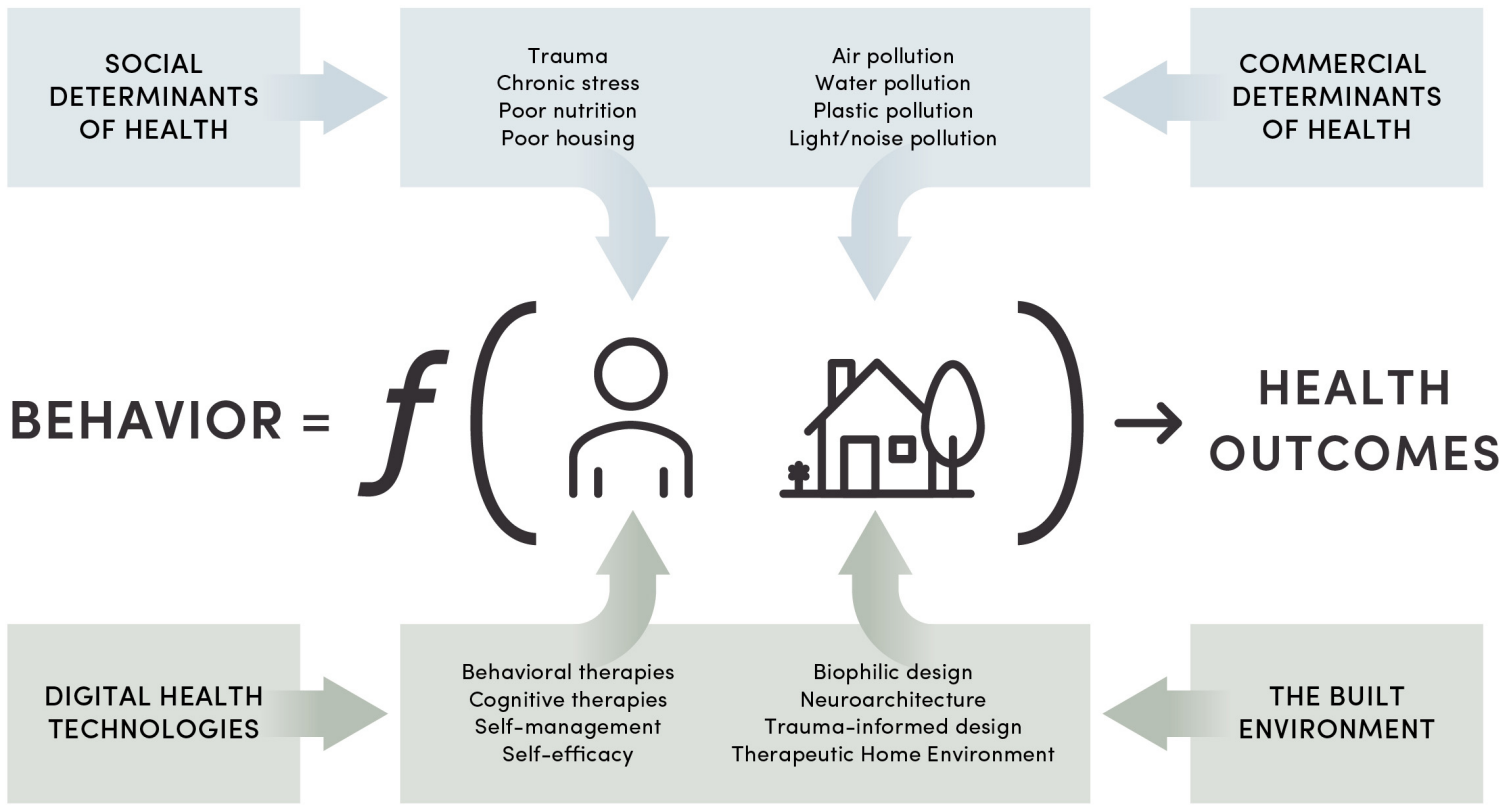
The applicability of the Kurt Lewin's field theory to integrating health behavior, digital health interventions and the built environment. The original theory, B = f (P,E), states that behavior (B) is a function of a person (P) and environment (E). The Lewin's Force Field approach to health behavior change shows competition between the health-harming exposome and therapeutic interventions that encompass a patient and the environment.

Figure 1 summarizes the complex interplay between health, individual behavior and environmental exposure. The exposome and a person's behavior are affected by both social and commercial determinants of health (56–58). Poor housing and nutrition, especially when coupled with environmental pollution, elevate the risk for a broad range of chronic conditions (59, 60). Furthermore, exposure to trauma and chronic stress significantly influence the onset and progression of chronic disease, as well as treatment outcomes (61–63). Based on research studies, we hypothesize that the built environment that fosters positive affect and self-management can influence health behavior, and thus health outcomes (64–66).

## 4 How can the built environment impact health outcomes?

The built environment (housing, workplaces, schools, healthcare facilities, neighborhoods etc) can have either negative or positive effects on individual and public health. Most people are aware of the environmental impact on health, as exemplified by air pollution, including indoor air quality (60, 67). Harvard's Healthy Buildings program offers educational information on optimizing housing and workplaces for health focused mostly on mitigating the negative effects of the built environment (68, 69). The expert tips on creating healthier homes include recommendations to reduce the exposure to unhealthy and toxic chemicals and to “(re)connect with nature and natural light indoors” (68). However, despite available information about toxic hazards associated with household products, consumer's willingness to pay for safer alternatives is low (70). For people living with conditions related to the nervous, neuroendocrine or immune systems, it is important to reduce the exposure to unhealthy household goods that may disrupt their functions (20).

In the book “Constructing Health,” Tye Farrow bridges translational research on enriched environment with designing spaces that can actively promote healing and health (71). The author describes examples of salutogenic architecture in order to create the built environment that provides neurological, psychological and emotional benefits. Grounded in the salutogenesis framework, salutogenic design aims to actively promote health by supporting an individual's “sense of coherence” through comprehensibility (easy navigation of spaces), manageability (providing a sense of control), and meaningfulness (spaces fostering a sense of purpose), collectively known as generalized resistance resources (24, 72). The patient-centered applications of salutogenic design include healthcare environments that support recovery and healing through connection with nature, provide social support, and offer opportunities for both relaxation and physical activity (73). Salutogenic spaces contribute to self-efficacy through cues that nurture confidence and relaxation (74).

The main objective of neuroarchitecture is to integrate neuroscience, environmental psychology and architecture in order to optimize design features, spatial arrangements, lighting, colors and acoustics for better health and wellbeing (25). A person's experience of neuroarchitecture-based environments modulates the activity of specific brain structures, such as amygdala, the prefrontal cortex, the anterior cingulate cortex, as well as the hypothalamic-pituitary-adrenal axis (75–78). Neuroarchitecture and neuroaesthetics research shows that intentionally-designed built environment can provide personal comfort, fascination and coherence, while yielding physiological, neurocognitive, behavioral and emotional responses (79–83). Physiological stress is also affected by architectural and nature-inspired interior features (81, 84–86).

Biophilic design is an approach to architecture and interior design that incorporates natural materials, patterns and elements into the built environment in order to reconnect humans with nature (23). Grounded in the biophilia and attention-restoration theories, biophilic design creates enriched environments that evoke multisensory experiences leading to restorative states and diverse physiological responses (87). Based on research studies, examples of the health-related benefits of biophilic design elements include: (*a*) stress reduction and improved recovery from stress, (*b*) improved positive emotions and mood, (*c*) reduced anxiety and depressive symptoms, (*d*) lowering blood pressure and heart rate, (*e*) improved pain management and cognitive functions, and (*f*) improved immune functions (87–90). Investigating biophilic intervention for cognitive functions in diabetic patients highlights the knowledge gap on long-term effects of biophilic design for chronically-ill patients (91).

Research on healthcare outcomes of biophilic design shows that the presence of biophilic features in hospitals can: (*a*) shorten the postsurgical recovery and hospitalization time, (*b*) reduce mortality and healthcare utilization (*c*) improve pain management, and (*e*) reduce stress for patients and healthcare professionals (92–94). Similarly, nature-enriched neighborhoods were shown to reduce healthcare utilization for mental and cardiovascular diseases (95–97). Based on emerging evidence and the ongoing research, biophilic design can be harnessed together with disease self-management to create therapeutic home environments for people living with chronic pain, migraine, depression, anxiety, cancer and other chronic conditions (20, 26).

Biophilic design is also recognized as a strategy to create therapeutic spaces for people living with dementia (98), Parkinson's disease (99), diabetes (91), and cancer survivors (100, 101). Medical applications of biophilic design can be illustrated by a “refuge and prospect” space intended to reduce stress, mitigate allostatic load and support cognitive reserve through rebalancing the autonomic nervous system and nurturing neuroplasticity. Biophilic attributes and neuroarchitecture can enhance response to analgesic, anxiolytic and antidepressant drugs, further supporting the relationships between intentionally-designed built environment and therapy outcomes (102). In conclusion, biophilic design offers unique prospects to transform the built environment into a therapeutic modality.

## 5 Integrating digital health and biophilic design

From translational point of view, the pleiotropic effects of biophilic design and the enriched environment can deliver broad-spectrum therapeutic effects, just like physical exercise, patient education and cognitive behavioral therapies (4, 20, 71). Therefore, transforming biophilic design into adjunctive therapies creates a novel value proposition for digital and pharma companies innovating medical treatments for chronic conditions. As detailed below, DHT are uniquely positioned to integrate biophilic design with behavioral, physical, and pharmacological interventions—enabling “enriched environment-enhanced” multimodal therapies.

**Figure 2:**
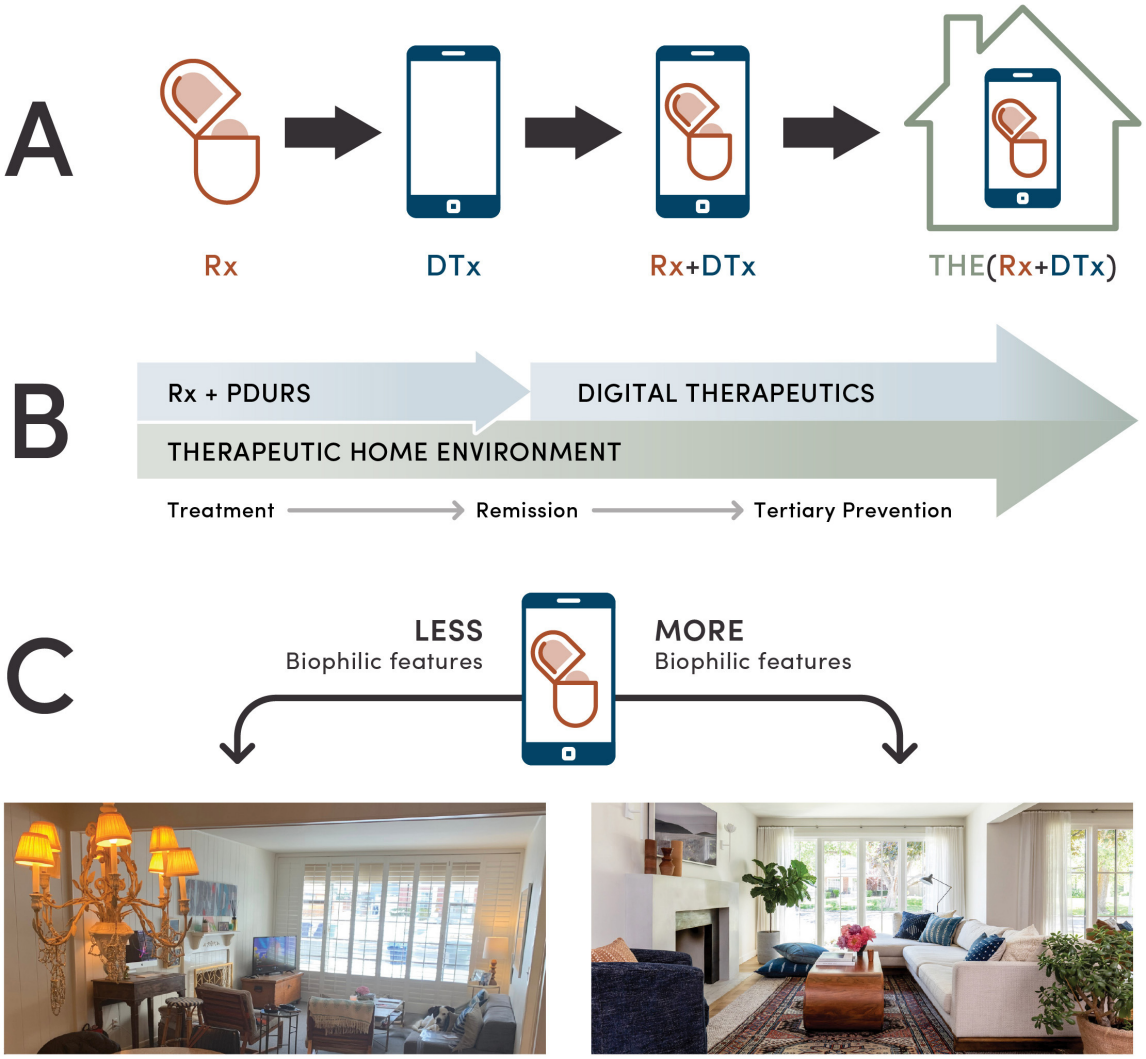
Integration of digital health interventions with a patient's home environment. **(A)** Evolving health technologies from prescription drugs (Rx) to digital therapeutics (DTx) and their integration with the therapeutic home environment (THE). **(B)** One of the roles of the therapeutic home environment in a long-term management of chronic conditions. Optimizing a patient home environment for accelerated healing, prevention of disease symptoms and a relapse illustrates the ability of the therapeutic home environment to improve digital health intervention outcomes beyond remission. **(C)** A hypothetical example of a drug + digital combination therapy for a chronic condition where a patient's home environment is optimized using biophilic design, offering multisensory engagement through natural light and other biophilic elements such as indoor plants, natural materials and patterns. Through biophilic features and optimized spatial arrangement, this redesigned living room fosters stress reduction, relaxation and self-efficacy.

Figure 2A shows the evolving role of DHT in chronic disease management. The emergence of DTx opened several strategies for the delivery of non-pharmacological therapies and their subsequent integration with prescription medications (41–43). In our earlier work, we advocated for leveraging digital technologies to amplify the therapeutic effects of music, physical activity, sleep hygiene, breathing exercises, mindfulness meditation, yoga, and other self-care practices, as well as integrating these aforementioned self-care practices with pharmacotherapies for depression, epilepsy, chronic pain, and cancer (103–105). More recently, we provided a rationale for developing multimodal interventions that combine DHT, prescription drugs and the therapeutic home environment (20).

One approach to integrate biophilic design and digital interventions is by delivering patient education focused on how natural environments provide health benefits and offering biophilic design DIY (do-it-yourself) actionable insights (20). This notion is further supported by biophilic VR interventions that showed positive effects such as stress reduction, alleviating anxiety and pain, and behavior change in oncology patients (106–109). Biophilic design is well positioned to enhance health outcomes of the smart home technologies that employ consumer electronics, wearables and mobile apps delivering health information (e.g., Samsung's “Home for Wellness” ecosystem), as previously suggested for the aging population (110–112). Emergence of AI-powered mobile apps and extended reality (XR) technologies for interior designers also illustrates opportunities to provide ideas for health-centric optimization of a patient's home environment (113). Another strategy to bridge the health benefits of biophilic design and digital health is through health-centric, household goods e-commerce as a digital health platform that enables creating the therapeutic home environment (20).

Promoting biophilic design through DHT can benefit pharma and biotech companies. For example, PDURS-based drug + digital combination therapy is an innovative approach to simultaneously treat chronic conditions at the molecular and behavioral levels. As illustrated in Figure 2B, once reaching remission, continuum of care through digital interventions reinforces the tertiary prevention. The therapeutic home environment may further improve the effectiveness of Rx+DTx combinations by accelerating remission of chronic pain, migraine, depression, anxiety or cancer (20). This aspect is illustrated in Figure 2C highlighting how living spaces that provide more natural light and other biophilic elements can integrate drug + digital + biophilic interventions. Once a patient reaches remission, deprescribing medications can be mitigated by a continuous use of DTx. In such cases, the therapeutic home environment that comprises biophilic spaces fostering self-care can support the prevention of relapses through restorative effects. For people living with epilepsy, such approach can improve the effectiveness of drug + digital combination therapy in controlling epileptic seizures (43).

## 6 Indications and future directions

Digital and pharmacological interventions for chronic diseases can be enhanced by biophilic, enriched environments intended to reduce stress, improve positive affect, rebalance the autonomic nervous system and promote neuroplasticity (20). For example, DTx-delivered Rhythmic Auditory Stimulation therapy for Parkinson's disease (PD) can be enhanced through multisensory home environment designed for PD patients (32, 99, 114, 115). To ameliorate the prognosis for mild cognitive impairment and dementia, DHT and biophilic design can simultaneously deliver multisensory experiences intended to improve patient care, including management of comorbidities, e.g., depression (98, 116–119). Such integrative approach also applies to enhancing outcomes of non-invasive sensory stimulation technologies and music-based interventions for the Alzheimer's disease (119–121). For chronic pain and migraine, outcomes of DTx and drug + digital combination therapies can be further improved in the presence of intentionally-designed biophilic home environment that fosters self-care practices (20, 26, 31, 122, 123). As emphasized in Figure 2C, drug + digital combination therapies for chronic pain or depression can be further optimized by the therapeutic home environment that offers multisensory stimulation through natural light and greenery, haptic feedback from natural materials, and personalized soundscapes that support positive emotions (124). Based on aforementioned examples, we conclude that home ecosystem can be embodied in AI-powered digital interventions to enhance overall patient experience, engagement and, ultimately, outcomes.

DHT are uniquely positioned to validate a patient's home environment as a therapeutic target for chronic disease prevention and treatment (20). Furthermore, incorporating biophilic design, as an “active non-pharmacological ingredient,” into digital therapies can improve their effectiveness, hence creating a new value proposition for digital health companies, healthcare systems and payers, to name a few examples of stakeholders. In our previous work, we described household goods e-commerce as a digital health platform delivering biophilic design and therapeutic home environment for specific chronic conditions (20), illustrating one possible research and development (R&D) strategy to integrate biophilic design and DHT. Another opportunity for advancing “enriched environment-enhanced” DTx solutions is expanding content by adding biophilic e-design functionality, biophilic design education focused on the health benefits, and incorporation of fractal designs into visuals to enhance user experience via relaxation (125, 126). For XR-based DHT, incorporating health-centric e-design features can integrate behavioral and environmental interventions. Given the growing interest in longevity and healthy aging, creating biophilic housing communities that prioritize connection with nature, social interactions and physical activities may also positively impact the effectiveness of digital interventions (127, 128).

## 7 Limitations

While this perspective article highlights opportunities to pivot DHT toward harnessing medicinal properties of biophilic design, we acknowledge real-world barriers for such approach. For example, the knowledge gap on long-term effects of biophilic interventions for chronic conditions, when combined with the knowledge-practice gap can delay a broader adoption of biophilic design into healthcare by many years. We suggest that the real-world pathway to validate biophilic design as a long-term therapeutic modality is through pragmatic clinical trials in hospitals, hospital at home programs, neurorehabilitation facilities, senior at-home care and assisted-living care settings (26, 92, 98, 129, 130). Testing the effects of biophilic design in improving patient's outcomes is feasible through remote patient monitoring employed in hospital at home programs (130, 131).

Additionally, a lack of awareness among general public and healthcare professionals about the impact of home environment on health outcomes poses a challenge to accelerate R&D activities to optimize health-centric solutions for a patient's living spaces. While limited return on investment (ROI) analysis for biophilic design are related to workplaces, education, hospitality and healthcare (132, 133), to the best of our knowledge, there are no health economics and outcomes research (HEOR) data on the use of biophilic design and a patient's home environment-based interventions for chronic diseases.

The commercial success of integrating digital health with biophilic design hinges on more than just innovation; it requires a nuanced understanding of market dynamics and user adoption. While the potential for improved health outcomes is promising, the real challenge lies in creating scalable, economically viable solutions that meet both consumer demand and organizational goals. DHT must not only complement natural environments and improve human health but be designed in ways that drive measurable ROI for companies—whether through improved outcomes, enhanced user engagement, productivity or reduced healthcare costs. Bridging the gap between these disciplines isn't just about technology or design; it's about creating sustainable business models that align with the evolving needs of consumers and the healthcare industry.

## 8 Conclusions

There are needs to improve the prevention and treatment outcomes for people living with chronic disorders, in particular for those who struggle with refractory conditions. Software-based health technologies have unique abilities to deliver multimodal therapies comprising cognitive and behavioral interventions, physical activities, patient education, disease self-management and self-efficacy. Given growing research evidence on medicinal properties of biophilic design and enriched environments, DHT are uniquely positioned to bridge interior design and health outcomes by delivering biophilic interventions, as well as integrating home environment with pharmacotherapies using the PDURS framework or adjunctive DTx strategy.

Digital technologies that transform the built environment into therapeutic spaces can benefit diverse stakeholders, including patients, healthcare professionals, value-based healthcare systems, payers, digital health and pharma companies, architects, interior designers, social impact and real estate investors. We hope that our perspective article will encourage DHT innovations to target chronic diseases at the combined person + behavior + environment levels. Our call to action is to initiate a dialogue on how to harness the medicinal properties of intentionally-designed spaces to enhance patient-centered care through digital health interventions.

## Data Availability

The original contributions presented in the study are included in the article/supplementary material, further inquiries can be directed to the corresponding author.

## References

[1] Patel NA Butte AJ. Characteristics and challenges of the clinical pipeline of digital therapeutics. NPJ Digit Med. (2020) 3:159. 10.1038/s41746-020-00370-8 33311567 PMC7733514

[2] Wang C Lee C Shin H. Digital therapeutics from bench to bedside. NPJ Digit Med. (2023) 6:38. 10.1038/s41746-023-00777-z 36899073 PMC10006069

[3] Chengyu Z Xueyan H Ying F. Research on disease management of chronic disease patients based on digital therapeutics: a scoping review. Digital Health. (2024) 10:20552076241297064. 10.1177/20552076241297064 39525556 PMC11544657

[4] Biskupiak Z Ha VV Rohaj A Bulaj G. Digital therapeutics for improving effectiveness of pharmaceutical drugs and biological products: preclinical and clinical studies supporting development of drug + digital combination therapies for chronic diseases. J Clin Med. (2024) 13:403. 10.3390/jcm13020403 38256537 PMC10816409

[5] Schmidt L Pawlitzki M Renard BY Meuth SG Masanneck L. The three-year evolution of Germany's digital therapeutics reimbursement program and its path forward. NPJ Digit Med. (2024) 7:139. 10.1038/s41746-024-01137-1 38789620 PMC11126413

[6] Watson A Chapman R Shafai G Maricich YA. FDA regulations and prescription digital therapeutics: evolving with the technologies they regulate. Front Digit Health. (2023) 5:1086219. 10.3389/fdgth.2023.1086219 37139487 PMC10150093

[7] van Kessel R Roman-Urrestarazu A Anderson M Kyriopoulos I Field S Monti G. Mapping factors that affect the uptake of digital therapeutics within health systems: scoping review. J Med Internet Res. (2023) 25:e48000. 10.2196/48000 37490322 PMC10410406

[8] Borges do Nascimento IJ Abdulazeem H Vasanthan LT Martinez EZ Zucoloto ML Østengaard L. Barriers and facilitators to utilizing digital health technologies by healthcare professionals. NPJ Digit Med. (2023) 6:161. 10.1038/s41746-023-00899-4 37723240 PMC10507089

[9] Gan DZQ McGillivray L Han J Christensen H Torok M. Effect of engagement with digital interventions on mental health outcomes: a systematic review and meta-analysis. Front Digit Health. (2021) 3:764079. 10.3389/fdgth.2021.764079 34806079 PMC8599127

[10] Nwosu A Boardman S Husain MM Doraiswamy PM. Digital therapeutics for mental health: is attrition the achilles heel? Front Psychiatry. (2022) 13:900615. 10.3389/fpsyt.2022.900615 35982936 PMC9380224

[11] Cho PJ Olaye IM Shandhi MMH Daza EJ Foschini L Dunn JP. Identification of key factors related to digital health observational study adherence and retention by data-driven approaches: an exploratory secondary analysis of two prospective longitudinal studies. Lancet Digital Health. (2025) 7:e23–34. 10.1016/S2589-7500(24)00219-X 39722250 PMC11725373

[12] Cheah KJ Abdul Manaf Z Fitri Mat Ludin A Razalli NH Mohd Mokhtar N Md Ali SH. Mobile apps for common noncommunicable disease management: systematic search in app stores and evaluation using the mobile app rating scale. JMIR Mhealth Uhealth. (2024) 12:e49055. 10.2196/49055 38532298 PMC11004629

[13] Argentieri MA Amin N Nevado-Holgado AJ Sproviero W Collister JA Keestra SM. Integrating the environmental and genetic architectures of aging and mortality. Nat Med. (2025) 31:1016–25. 10.1038/s41591-024-03483-9 39972219 PMC11922759

[14] Münzel T Sørensen M Hahad O Nieuwenhuijsen M Daiber A. The contribution of the exposome to the burden of cardiovascular disease. Nat Rev Cardiol. (2023) 20:651–69. 10.1038/s41569-023-00873-3 37165157

[15] Ward JS Duncan JS Jarden A Stewart T. The impact of children's exposure to greenspace on physical activity, cognitive development, emotional wellbeing, and ability to appraise risk. Health Place. (2016) 40:44–50. 10.1016/j.healthplace.2016.04.015 27179137

[16] De la Fuente F Saldías MA Cubillos C Mery G Carvajal D Bowen M. Green space exposure association with type 2 diabetes mellitus, physical activity, and obesity: a systematic review. Int J Environ Res Public Health. (2021) 18:97. 10.3390/ijerph18010097 33375559 PMC7796153

[17] Wilson B Neale C Roe J. Urban green space access, social cohesion, and mental health outcomes before and during COVID-19. Cities. (2024) 152:105173. 10.1016/j.cities.2024.105173

[18] Walton L Skillen E Mosites E Bures RM Amah-Mbah C Sandoval M. The intersection of health and housing: analysis of the research portfolios of the National Institutes of Health, Centers for Disease Control and Prevention, and U.S department of housing and urban development. PLoS ONE. (2024) 19:e0296996. 10.1371/journal.pone.0296996 38285706 PMC10824422

[19] Ghorbany S Hu M Yao S Sisk M Wang C Zhang K. Data driven assessment of built environment impacts on urban health across United States cities. Sci Rep. (2025) 15:19998. 10.1038/s41598-025-04567-3 40481030 PMC12144143

[20] Huntsman DD Bulaj G. Home Environment as a therapeutic target for prevention and treatment of chronic diseases: delivering restorative living spaces, patient education and self-care by bridging biophilic design, e-commerce and digital health technologies. Int J Environ Res Public Health. (2025) 22:225. 10.3390/ijerph22020225 40003451 PMC11855921

[21] Hernandez-Garcia E Chrysikou E Nekhlyudov L Gilroy DW Ordóñez-Mena JM. Home-built environment interventions and inflammation biomarkers: a systematic review and meta-analysis protocol. BJGP Open. (2022) 6:BJGPO.2022.0104. 10.3399/BJGPO.2022.0104 36137647 PMC9904785

[22] Hernandez-Garcia E Chrysikou E Kalea AZ. The interplay between housing environmental attributes and design exposures and psychoneuroimmunology profile—an exploratory review and analysis paper in the cancer survivors' mental health morbidity context. Int J Environ Res Public Health. (2021) 18:10891. 10.3390/ijerph182010891 34682637 PMC8536084

[23] Kellert SR Heerwagen J Mador M. Biophilic Design: The Theory, Science, and Practice of Bringing Buildings to Life. Hoboken, N.J.: Wiley (2008).

[24] Dilani A. Psychosocially supportive design: a salutogenic approach to the design of the physical environment. Des Health Sci Rev. (2008) 1:47–55.

[25] Wang S Sanches de Oliveira G Djebbara Z Gramann K. The embodiment of architectural experience: a methodological perspective on neuro-architecture. Front Hum Neurosci. (2022) 16:833528. 10.3389/fnhum.2022.833528 35615743 PMC9124889

[26] Huntsman DD Bulaj G. Healthy dwelling: design of biophilic interior environments fostering self-care practices for people living with migraines, chronic pain, and depression. Int J Environ Res Public Health. (2022) 19:2248. 10.3390/ijerph19042248 35206441 PMC8871637

[27] Ribba B Peck R Hutchinson L Bousnina I Motti D. Digital therapeutics as a new therapeutic modality: a review from the perspective of clinical pharmacology. Clin Pharmacol Ther. (2023) 114:578–90. 10.1002/cpt.2989 37392464

[28] Garcia LM Birckhead BJ Krishnamurthy P Sackman J Mackey IG Louis RG. An 8-week self-administered at-home behavioral skills-based virtual reality program for chronic low back pain: double-blind, randomized, placebo-controlled trial conducted during COVID-19. J Med Internet Res. (2021) 23:e26292. 10.2196/26292 33484240 PMC7939946

[29] Shomali M Mora P Aleppo G Peeples M Kumbara A MacLeod J. The critical elements of digital health in diabetes and cardiometabolic care. Front Endocrinol. (2024) 15:1469471. 10.3389/fendo.2024.1469471 39351525 PMC11439689

[30] Gussoni G Ravot E Zecchina M Recchia G Santoro E Ascione R. Digital therapeutics in oncology: findings, barriers and prospects. A narrative review. Ann Res Oncol. (2022) 2:55–69. 10.48286/aro.2022.39

[31] Abbadessa G Brigo F Clerico M De Mercanti S Trojsi F Tedeschi G. Digital therapeutics in neurology. J Neurol. (2022) 269:1209–24. 10.1007/s00415-021-10608-4 34018047 PMC8136262

[32] Ellis TD Earhart GM. Digital therapeutics in Parkinson's disease: practical applications and future potential. J Parkinsons Dis. (2021) 11:S95–S101. 10.3233/JPD-202407 33646177 PMC8292155

[33] Philippe TJ Sikder N Jackson A Koblanski ME Liow E Pilarinos A. Digital health interventions for delivery of mental health care: systematic and comprehensive meta-review. JMIR Ment Health. (2022) 9:e35159. 10.2196/35159 35551058 PMC9109782

[34] Moschonis G Siopis G Jung J Eweka E Willems R Kwasnicka D. Effectiveness, reach, uptake, and feasibility of digital health interventions for adults with type 2 diabetes: a systematic review and meta-analysis of randomised controlled trials. Lancet Digital Health. (2023) 5:e125–e43. 10.1016/S2589-7500(22)00233-3 36828606

[35] Solomon DH Rudin RS. Digital health technologies: opportunities and challenges in rheumatology. Nat Rev Rheumatol. (2020) 16:525–35. 10.1038/s41584-020-0461-x 32709998

[36] Spatz ES Ginsburg GS Rumsfeld JS Turakhia MP. Wearable digital health technologies for monitoring in cardiovascular medicine. N Engl J Med. (2024) 390:346–56. 10.1056/NEJMra2301903 38265646

[37] Doherty C Baldwin M Keogh A Caulfield B Argent R. Keeping pace with wearables: a living umbrella review of systematic reviews evaluating the accuracy of consumer wearable technologies in health measurement. Sports Med. (2024) 54:2907–26. 10.1007/s40279-024-02077-2 39080098 PMC11560992

[38] Hirten RP Danieletto M Sanchez-Mayor M Whang JK Lee KW Landell K. Physiological data collected from wearable devices identify and predict inflammatory bowel disease flares. Gastroenterology. (2025) 168:939–51.e5. 10.1053/j.gastro.2024.12.024 39826619 PMC12206379

[39] Butler PM Yang J Brown R Hobbs M Becker A Penalver-Andres J. Smartwatch- and smartphone-based remote assessment of brain health and detection of mild cognitive impairment. Nat Med. (2025) 31:829–39. 10.1038/s41591-024-03475-9 40038507 PMC11922773

[40] Zheng NS Annis J Master H Han L Gleichauf K Ching JH. Sleep patterns and risk of chronic disease as measured by long-term monitoring with commercial wearable devices in the all of US research program. Nat Med. (2024) 30:2648–56. 10.1038/s41591-024-03155-8 39030265 PMC11405268

[41] Bulaj G. Combining non-pharmacological treatments with pharmacotherapies for neurological disorders: a unique interface of the brain, drug-device, and intellectual property. Front Neurol. (2014) 5:126. 10.3389/fneur.2014.00126 25071711 PMC4095562

[42] Sverdlov O van Dam J Hannesdottir K Thornton-Wells T. Digital therapeutics: an integral component of digital innovation in drug development. Clin Pharmacol Ther. (2018) 104:72–80. 10.1002/cpt.1036 29377057

[43] Afra P Bruggers CS Sweney M Fagatele L Alavi F Greenwald M. Mobile software as a medical device (SaMD) for the treatment of epilepsy: development of digital therapeutics comprising behavioral and music-based interventions for neurological disorders. Front Hum Neurosci. (2018) 12:171. 10.3389/fnhum.2018.00171 29780310 PMC5946004

[44] FDA. Regulatory Considerations for Prescription Drug Use-Related Software: FDA. (2023). Available online at: https://www.fda.gov/regulatory-information/search-fda-guidance-documents/regulatory-considerations-prescription-drug-use-related-software (Accessed June 29, 2025).

[45] Sheeran P Klein WMP Rothman AJ. Health behavior change: moving from observation to intervention. Annu Rev Psychol. (2017) 68, 573–600. 10.1146/annurev-psych-010416-044007 27618942

[46] Taj F Klein MCA van Halteren A. Digital health behavior change technology: bibliometric and scoping review of two decades of research. JMIR Mhealth Uhealth. (2019) 7:e13311. 10.2196/13311 31833836 PMC6935048

[47] Owen C Crane J. Trauma-informed design of supported housing: a scoping review through the lens of neuroscience. Int J Environ Res Public Health. (2022) 19:14279. 10.3390/ijerph192114279 36361166 PMC9658651

[48] Elser H Kruse CFG Schwartz BS Casey JA. The environment and headache: a narrative review. Curr Environ Health Rep. (2024) 11:184–203. 10.1007/s40572-024-00449-4 38642284

[49] Friedman DI De Ver Dye T. Migraine and the environment. Headache. (2009) 49:941–52. 10.1111/j.1526-4610.2009.01443.x 19545255

[50] Agbetou M Adoukonou T. Lifestyle modifications for migraine management. Front Neurol. (2022) 13:719467. 10.3389/fneur.2022.719467 35370920 PMC8971279

[51] Riva A Rebecchi A Capolongo S Gola M. Can homes affect well-being? A scoping review among housing conditions, indoor environmental quality, and mental health outcomes. Int J Environ Res Public Health. (2022) 19:15975. 10.3390/ijerph192315975 36498051 PMC9736414

[52] Frank LD Adhikari B White KR Dummer T Sandhu J Demlow E. Chronic disease and where you live: built and natural environment relationships with physical activity, obesity, and diabetes. Environ Int. (2022) 158:106959. 10.1016/j.envint.2021.106959 34768046

[53] Nguyen QC Tasdizen T Alirezaei M Mane H Yue X Merchant JS. Neighborhood built environment, obesity, and diabetes: a Utah siblings study. SSM—Population Health. (2024) 26:101670. 10.1016/j.ssmph.2024.101670 38708409 PMC11068633

[54] Brug J van Lenthe FJ Kremers SPJ. Revisiting Kurt Lewin: how to gain insight into environmental correlates of obesogenic behaviors. Am J Prev Med. (2006) 31:525–9. 10.1016/j.amepre.2006.08.016 17169715

[55] Beulens JWJ Pinho MGM Abreu TC den Braver NR Lam TM Huss A. Environmental risk factors of type 2 diabetes—an exposome approach. Diabetologia. (2022) 65:263–74. 10.1007/s00125-021-05618-w 34792619

[56] Hacker K Thomas CW Zhao G Claxton JS Eke P Town M. Social determinants of health and health-related social needs among adults with chronic diseases in the united states, behavioral risk factor surveillance system, 2022. Prev Chronic Dis. (2024) 21:E94. 10.5888/pcd21.240362 39602222 PMC11608007

[57] Hill-Briggs F Adler NE Berkowitz SA Chin MH Gary-Webb TL Navas-Acien A. Social determinants of health and diabetes: a scientific review. Diabetes Care. (2020) 44:258–79. 10.2337/dci20-0053 33139407 PMC7783927

[58] Mialon M. An overview of the commercial determinants of health. Global Health. (2020) 16:74. 10.1186/s12992-020-00607-x 32807183 PMC7433173

[59] Krieger J Higgins DL. Housing and health: time again for public health action. Am J Public Health. (2002) 92:758–68. 10.2105/AJPH.92.5.758 11988443 PMC1447157

[60] Bennitt FB Wozniak SS Causey K Burkart K Brauer M. Estimating disease burden attributable to household air pollution: new methods within the Global Burden of Disease Study. Lancet Global Health. (2021) 9:S18. 10.1016/S2214-109X(21)00126-1

[61] Antoniou G Lambourg E Steele JD Colvin LA. The effect of adverse childhood experiences on chronic pain and major depression in adulthood: a systematic review and meta-analysis. Br J Anaesth. (2023) 130:729–46. 10.1016/j.bja.2023.03.008 37087334 PMC10251130

[62] Sonu S Post S Feinglass J. Adverse childhood experiences and the onset of chronic disease in young adulthood. Prev Med. (2019) 123:163–70. 10.1016/j.ypmed.2019.03.032 30904602

[63] Tidmarsh LV Harrison R Ravindran D Matthews SL Finlay KA. The influence of adverse childhood experiences in pain management: mechanisms, processes, and trauma-informed care. Front Pain Res. (2022) 3:923866. 10.3389/fpain.2022.923866 35756908 PMC9226323

[64] Shiota MN Papies EK Preston SD Sauter DA. Positive affect and behavior change. Curr Opin Behav Sci. (2021) 39:222–8. 10.1016/j.cobeha.2021.04.022

[65] Kondo MC Fluehr JM McKeon T Branas CC. Urban green space and its impact on human health. Int J Environ Res Public Health. (2018) 15:445. 10.3390/ijerph15030445 29510520 PMC5876990

[66] Hunter RF Christian H Veitch J Astell-Burt T Hipp JA Schipperijn J. The impact of interventions to promote physical activity in urban green space: a systematic review and recommendations for future research. Soc Sci Med. (2015) 124:246–56. 10.1016/j.socscimed.2014.11.051 25462429

[67] Allen JG. Recommitting to ventilation standards for healthy indoor air quality. Am J Public Health. (2024) 114:991–3. 10.2105/AJPH.2024.307809 39231402 PMC11375373

[68] Allen JG Cedeno-Laurent J Jones E Luna M Macnaughton P Robinson S. Homes for Health: 36 Expert Tips to Make Your Home A Healthier Home. Harvard TH Chan School of Public Health (2019). p. 28.

[69] Allen JG Bernstein A Cao X Eitland E Flanigan S Gokhale M. The 9 Foundations of A Healthy Building. Harvard: School of Public Health (2017).

[70] Boman A Miguel M Andersson I Slunge D. The effect of information about hazardous chemicals in consumer products on behaviour—a systematic review. Sci Total Environ. (2024) 947:174774. 10.1016/j.scitotenv.2024.174774 39009144

[71] Farrow T. Constructing Health: How the Built Environment Enhances Your Mind's Health. Toronto: University of Toronto Press (2024). 10.3138/9781487557232

[72] Antonovsky A. The salutogenic model as a theory to guide health promotion. Health Promot Int. (1996) 11:11–8. 10.1093/heapro/11.1.11

[73] Golembiewski JA. “Salutogenic architecture in healthcare settings.” In: *The Handbook of Salutogenesis*. (2017). p. :267–76. 10.1007/978-3-319-04600-6_26 28590652

[74] Mazzi A. Toward a unified language (and application) of salutogenic design: an opinion paper. HERD. (2021) 14:337–49. 10.1177/1937586720967347 33124468

[75] Medhat Assem H Mohamed Khodeir L Fathy F. Designing for human wellbeing: the integration of neuroarchitecture in design—a systematic review. Ain Shams Eng J. (2023) 14:102102. 10.1016/j.asej.2022.102102

[76] Banaei M Hatami J Yazdanfar A Gramann K. Walking through architectural spaces: the impact of interior forms on human brain dynamics. Front Hum Neurosci. (2017) 11:477. 10.3389/fnhum.2017.00477 29033807 PMC5627023

[77] Abbas S Okdeh N Roufayel R Kovacic H Sabatier J-M Fajloun Z. Neuroarchitecture: how the perception of our surroundings impacts the brain. Biology. (2024) 13:220. 10.3390/biology13040220 38666832 PMC11048496

[78] Valentine C Mitcheltree H Sjövall IAK Khalil MH. Architecturally mediated allostasis and neurosustainability: a proposed theoretical framework for the impact of the built environment on neurocognitive health. Brain Sci. (2025) 15:201. 10.3390/brainsci15020201 40002534 PMC11853682

[79] Higuera-Trujillo JL Llinares C Macagno E. The cognitive-emotional design and study of architectural space: a scoping review of neuroarchitecture and its precursor approaches. Sensors. (2021) 21:2193. 10.3390/s21062193 33801037 PMC8004070

[80] Chatterjee A Coburn A Weinberger A. The neuroaesthetics of architectural spaces. Cogn Process. (2021) 22:115–20. 10.1007/s10339-021-01043-4 34448969

[81] Valentine C. The impact of architectural form on physiological stress: a systematic review. Front Comput Sci. (2024) 5:1237531. 10.3389/fcomp.2023.123753127819847

[82] Valentine C Steffert T Mitcheltree H Steemers K. Architectural neuroimmunology: a pilot study examining the impact of biophilic architectural design on neuroinflammation. Buildings. (2024) 14:1292. 10.3390/buildings14051292

[83] Sussman A Hollander J. Cognitive Architecture: Designing for How we Respond to the Built Environment: New York, NY: Routledge (2021). 10.4324/978100303154336532166

[84] Fich LB Jönsson P Kirkegaard PH Wallergård M Garde AH Hansen Å. Can architectural design alter the physiological reaction to psychosocial stress? A virtual TSST experiment. Physiol Behav. (2014) 135:91–7. 10.1016/j.physbeh.2014.05.034 24907691

[85] Guan H Zhang X Dong J Shu R Hu S Tong Z. Biophilic environment with auditory-olfactory stimuli contributes to psychophysiological restoration from stress. Build Environ. (2025) 275:112830. 10.1016/j.buildenv.2025.112830

[86] McSweeney J Johnson S Sherry S Singleton J Rainham D. Indoor nature exposure and influence on physiological stress markers. Int J Environ Health Res. (2021) 31:636–50. 10.1080/09603123.2019.1679357 31625764

[87] Browning WD Ryan CO Clancy JO. “14 patterns of biophilic design.” In: Improving Health and Wellbeing in the Built Environment (10th Anniversary Edition). New York, NY: Terrapin Bright Green LLC (2024).

[88] Hung S-H Chang C-Y. Health benefits of evidence-based biophilic-designed environments: a review. J People Plants Environ. (2021) 24:1–16. 10.11628/ksppe.2021.24.1.1

[89] Zhong W Schröder T Bekkering J. Biophilic design in architecture and its contributions to health, well-being, and sustainability: a critical review. Front Archit Res. (2022) 11:114–41. 10.1016/j.foar.2021.07.006

[90] Gillis K Gatersleben B. A review of psychological literature on the health and wellbeing benefits of biophilic design. Buildings. (2015) 5:948–63. 10.3390/buildings503094840414183

[91] Dai J Wang M Zhang H Wang Z Meng X Sun Y. Effects of indoor biophilic environments on cognitive function in elderly patients with diabetes: study protocol for a randomized controlled trial. Front Psychol. (2025) 16:1512175. 10.3389/fpsyg.2025.1512175 40045967 PMC11880250

[92] Tekin BH Corcoran R Gutiérrez RU. A systematic review and conceptual framework of biophilic design parameters in clinical environments. HERD. (2023) 16:233–50. 10.1177/19375867221118675 35996349 PMC9755679

[93] Guidolin K Jung F Hunter S Yan H Englesakis M Verderber S. The influence of exposure to nature on inpatient hospital stays: a scoping review. HERD. (2024) 17:360–75. 10.1177/19375867231221559 38288612 PMC11080386

[94] Al Khatib I Samara F Ndiaye M. A systematic review of the impact of therapeutical biophilic design on health and wellbeing of patients and care providers in healthcare services settings. Front Built Environ. (2024) 10:1467692. 10.3389/fbuil.2024.1467692

[95] Makram OM Pan A Maddock JE Kash BA. Nature and mental health in urban Texas: a naturescore-based study. Int J Environ Res Public Health. (2024) 21:168. 10.3390/ijerph21020168 38397658 PMC10887946

[96] Keith RJ Hart JL Bhatnagar A. Greenspaces and cardiovascular health. Circ Res. (2024) 134:1179–96. 10.1161/CIRCRESAHA.124.323583 38662868 PMC12208525

[97] Bianconi A Longo G Coa AA Fiore M Gori D. Impacts of Urban green on cardiovascular and cerebrovascular diseases—a systematic review and meta-analysis. Int J Environ Res Public Health. (2023) 20:5966. 10.3390/ijerph20115966 37297570 PMC10253108

[98] Peters T Verderber S. Biophilic design strategies in long-term residential care environments for persons with dementia. J Aging Environ. (2021) 36:1–29. 10.1080/26892618.2021.1918815

[99] Hesam Shariati F Steffens A Adhami S. Designing environments that contribute to a reduction in the progression of Parkinson's disease; a literature review. Health Place. (2023) 83:103105. 10.1016/j.healthplace.2023.103105 37703785

[100] Ibrahim T Gabr H Khodeir L Aboubakr D. Synergetic approach for biophilic healing interior design for paediatric cancer. J Eng Appl Sci. (2020) 67:1435–53.

[101] Tekin BH Urbano Gutiérrez R. Human-centred health-care environments: a new framework for biophilic design. Front Med Technol. (2023) 5:1219897. 10.3389/fmedt.2023.1219897 37560462 PMC10408300

[102] Chiamulera C Benvegnù G Piva A Paolone G. Ecocebo: How the interaction between environment and drug effects may improve pharmacotherapy outcomes. Neurosci Biobehav Rev. (2024) 161:105648. 10.1016/j.neubiorev.2024.105648 38565340

[103] Bulaj G Ahern MM Kuhn A Judkins ZS Bowen RC Chen Y. Incorporating natural products, pharmaceutical drugs, self-care and digital/mobile health technologies into molecular-behavioral combination therapies for chronic diseases. Curr Clin Pharmacol. (2016) 11:128–45. 10.2174/1574884711666160603012237 27262323 PMC5011401

[104] Bulaj G Clark J Ebrahimi M Bald E. From precision metapharmacology to patient empowerment: delivery of self-care practices for epilepsy, pain, depression and cancer using digital health technologies. Front Pharmacol. (2021) 12:612602. 10.3389/fphar.2021.612602 33972825 PMC8105510

[105] Schriewer K Bulaj G. Music streaming services as adjunct therapies for depression, anxiety, and bipolar symptoms: convergence of digital technologies, mobile apps, emotions, and global mental health. Front Public Health. (2016) 4:217. 10.3389/fpubh.2016.00217 27747209 PMC5043262

[106] Ashley Verzwyvelt L McNamara A Xu X Stubbins R. Effects of virtual reality v. biophilic environments on pain and distress in oncology patients: a case-crossover pilot study. Sci Rep. (2021) 11:20196. 10.1038/s41598-021-99763-2 34642416 PMC8511009

[107] Luo W Chen C Zhou W Cao A Zhu W Zhou Y. Biophilic virtual reality on children's anxiety and pain during circumcision: a randomized controlled study. J Pediatr Urol. (2023) 19:201–10. 10.1016/j.jpurol.2022.10.023 36336624

[108] Yin J Yuan J Arfaei N Catalano PJ Allen JG Spengler JD. Effects of biophilic indoor environment on stress and anxiety recovery: a between-subjects experiment in virtual reality. Environ Int. (2020) 136:105427. 10.1016/j.envint.2019.105427 31881421

[109] Lee E-J Park S-J Choi J-H. Effect of a virtual biophilic residential environment on the perception and responses of seniors. Appl Sci. (2024) 14:11431. 10.3390/app142311431

[110] Lee EJ Park SJ A. Framework of smart-home service for elderly's biophilic experience. Sustainability. (2020) 12:8572. 10.3390/su12208572

[111] Lee E-J Park S-J. A preference-driven smart home service for the elderly's biophilic experience. Sensors. (2021) 21:5108. 10.3390/s21155108 34372342 PMC8348804

[112] Chrysikou E Biddulph JP Loizides F Savvopoulou E Rehn-Groenendijk J Jones N. Creating resilient smart homes with a heart: sustainable, technologically advanced housing across the lifespan and frailty through inclusive design for people and their robots. Sustainability. (2024) 16:5837. 10.3390/su16145837

[113] Cuevas JRG Rili JKR Santillan ANG Agustin VA Gonzales MGN Centeno CJ. “Layout loud: an ai-powered augmented reality and mobile application for room interior design and layout optimization.” In: 2024 International Conference on Intelligent Cybernetics Technology & Applications (ICICyTA). Ubud: IEEE (2024). 10.1109/ICICYTA64807.2024.10912928

[114] Zajac JA Porciuncula F Cavanaugh JT McGregor C Harris BA Smayda KE. Feasibility and proof-of-concept of delivering an autonomous music-based digital walking intervention to persons with Parkinson's disease in a naturalistic setting. J Parkinsons Dis. (2023) 13:1253–65. 10.3233/JPD-230169 37840504 PMC10657706

[115] Ottosson J Lavesson L Pinzke S Grahn P. The significance of experiences of nature for people with Parkinson's disease, with special focus on freezing of gait—the necessity for a biophilic environment. A multi-method single subject study. Int J Environ Res Public Health. (2015) 12:7274–99. 10.3390/ijerph120707274 26132480 PMC4515656

[116] Booher SG. Investigation of Biophilic Interventions to Improve Mood and Behavior of Persons with Dementia. Columbus, OH: The Ohio State University (2020).

[117] Di Lorito C Bosco A Rai H Craven M McNally D Todd C. A systematic literature review and meta-analysis on digital health interventions for people living with dementia and Mild Cognitive Impairment. Int J Geriatr Psychiatry. (2022) 37:5730. 10.1002/gps.5730 35588315 PMC9321868

[118] Park H Ha J. Effect of digital technology interventions for cognitive function improvement in mild cognitive impairment and dementia: a systematic review and meta-analysis. Res Nurs Health. (2024) 47:409–22. 10.1002/nur.22383 38567389

[119] Park JM Tsai L-H. Innovations in noninvasive sensory stimulation treatments to combat Alzheimer's disease. PLoS Biol. (2025) 23:e3003046. 10.1371/journal.pbio.3003046 40019895 PMC11870349

[120] Hajós M Boasso A Hempel E Shpokayte M Konisky A Seshagiri CV. Safety, tolerability, and efficacy estimate of evoked gamma oscillation in mild to moderate Alzheimer's disease. Front Neurol. (2024) 15:1343588. 10.3389/fneur.2024.1343588 38515445 PMC10957179

[121] Cheng Z Minmin Z Sabran K. Mobile app-based interventions to improve the well-being of people with dementia: a systematic literature review. Assist Technol. (2024) 36:64–74. 10.1080/10400435.2023.2206439 37115814

[122] Martin LF Patwardhan AM Jain SV Salloum MM Freeman J Khanna R. Evaluation of green light exposure on headache frequency and quality of life in migraine patients: a preliminary one-way cross-over clinical trial. Cephalalgia. (2021) 41:135–47. 10.1177/0333102420956711 32903062 PMC8034831

[123] Main A McCartney H Ibrar M Muirhead F Mavroeidi A Rai HK. Patients' experiences of digital health interventions for the self-management of chronic pain: systematic review and thematic synthesis. J Med Internet Res. (2025) 27:e69100. 10.2196/69100 40101209 PMC11962327

[124] Kimura LF Novaes LS Picolo G Munhoz CD Cheung CW Camarini R. How environmental enrichment balances out neuroinflammation in chronic pain and comorbid depression and anxiety disorders. Br J Pharmacol. (2022) 179:1640–60. 10.1111/bph.15584 34076891

[125] Taylor RP. The potential of biophilic fractal designs to promote health and performance: a review of experiments and applications. Sustainability. (2021) 13:823. 10.3390/su13020823

[126] Robles KE Roberts M Viengkham C Smith JH Rowland C Moslehi S. Aesthetics and psychological effects of fractal based design. Front Psychol. (2021) 12:699962. 10.3389/fpsyg.2021.699962 34484047 PMC8416160

[127] Milliken S Kotzen B Walimbe S Coutts C Beatley T. Biophilic cities and health. Cities Health. (2023) 7:175–88. 10.1080/23748834.2023.2176200

[128] Chum K Fitzhenry G Robinson K Murphy M Phan D Alvarez J. Examining community-based housing models to support aging in place: a scoping review. Gerontologist. (2020) 62:e178–e92. 10.1093/geront/gnaa142 32971538

[129] Miller E Burton LO. Redesigning aged care with a biophilic lens: a call to action. Cities Health. (2023) 7:260–72. 10.1080/23748834.2020.1772557

[130] Pandit JA Pawelek JB Leff B Topol EJ. The hospital at home in the USA: current status and future prospects. NPJ Digit Med. (2024) 7:48. 10.1038/s41746-024-01040-9 38413704 PMC10899639

[131] Whitehead D Conley J. The next frontier of remote patient monitoring: hospital at home. J Med Internet Res. (2023) 25:e42335. 10.2196/42335 36928088 PMC10132045

[132] Ryan CO Browning WD Walker DB. The Economics of Biophilia: Why Designing With Nature in Mind Makes Financial Sense. 2nd Ed. New York: Terrapin Bright Green (2023).

[133] Jana S. derlund, Peter N. Biophilic architecture: a review of the rationale and outcomes. AIMS Environ Sci. (2015) 2:950–69. 10.3934/environsci.2015.4.950

